# SCORE: Serologic evidence of COVID-19 and social and occupational contacts in healthcare workers in long-term care and acute care facilities in Southeastern Ontario (SCORE)

**DOI:** 10.1371/journal.pone.0303813

**Published:** 2025-08-13

**Authors:** Jorge L. Martinez-Cajas, Beatriz Alvarado, Ann Jolly, Yanping Gong, Bradley Stoner, Gerald Evans, Santiago Perez-Patrigeon, T. Hugh Guan

**Affiliations:** 1 Division of Infectious Diseases, Department of Medicine, Queen’s University, Kingston, Ontario, Canada; 2 Department of Public Health Sciences. Queen’s University, Kingston, Ontario, Canada; 3 Ottawa Public Health, Ottawa, Ontario, Canada; 4 Department of Pathology and Molecular Medicine, Queen’s University, Kingston, Ontario, Canada; 5 South East Health Unit (formerly KFL&A Public Health), Kingston, Ontario, Canada; Action Research Bangladesh, BANGLADESH

## Abstract

**Introduction:**

We established a longitudinal cohort of healthcare workers (HCWs) in an acute care hospital (ACH) and four long-term care homes (LTCHs) in Ontario, Canada, to follow the incidence of SARS-CoV-2 infection, humoral immune response to infection and/or vaccination, and determinants of infection risk. Here, we 1) describe the cohort regarding the distribution of main exposures, outcomes and serologic assays, 2) describe the unadjusted incidence of SARS-CoV-2 infection risk in the overall population, and 3) summarize the analysis and its pertinence.

**Methods and participants:**

HCWs were recruited between November 24, 2020, and July 24, 2021. They completed a baseline survey, monthly surveillance for 9–12 months, a post-Omicron-wave survey, and provided blood samples for anti-SARS-CoV-2 antibody measurements. We collected data on host-related (humoral response to vaccines and SARS-CoV-2 infection) and environmental factors (social contact history and occupational, household and community conditions). Descriptive analysis by setting, comparison of distributions, and unadjusted survival analysis were performed.

**Results:**

In total, 143 HCWs from the ACH and 57 from LTCHs had complete data, and 72% were followed until September 2022. Nearly 60% of the sample consisted of nurses, nurse assistants and personal support workers. Survival analysis showed that the risk of infection was bimodal, with low risk throughout the study period until the first Omicron wave. ACH HCWs had a higher risk of infection during the Omicron waves than during the preceding waves (Odds Ratio = 7.64; CI95%: 4.24–13.7), while LTCH HCWs at high-risk facilities experienced a similar risk of infection before and during the Omicron waves (OR = 1.76; CI95%: 0.63–4.9). During the Omicron waves, the use of protective equipment by HCWs working with institutional COVID-19 cases increased, but the use of community protective measures diminished. Household infections reported by participating HCWs also increased during the Omicron waves compared to previous waves. Immunoglobulin G (IgG) antibody levels increased over two time periods, (Pre vs Post- Omicron) likely due to the immune response to high levels of both vaccination and SARS-CoV-2 infections.

**Discussion:**

We observed a low incidence of COVID-19 until the onset of the Omicron waves, which highlights the drastic impact of this Variants of Concern (VOC) on transmission and the importance of infectious agent characteristics. Our analysis indicated a ninefold increased risk of infection compared to that in earlier pandemic periods. Further analysis will allow the estimation of 1) the risk factors for SARS-CoV-2 infection at the community, household and healthcare facility levels, 2) the relationship between humoral responses and SARS-CoV-2 infection/vaccination, and 3) the role of social contact in work, household and community settings in the risk of infection.

## 1. Introduction

Healthcare workers (HCWs) were at the forefront of the response to the Coronavirus disease (COVID-19)pandemic and are an essential societal resource in such situations. COVID-19 in HCWs weakened the healthcare system’s capacity to respond to the pandemic by causing absenteeism. Before the development of effective vaccines, HCWs relied solely on physical distancing, personal protective equipment (PPE) and institutional infection control protocols to avoid acquiring COVID-19 and limiting its spread. HCWs continued to care for COVID-19 patients despite their concern about the effectiveness of available PPE (surgical masks in 2020) in preventing SARS-CoV-2 infection. HCWs accounted for 19.4% of SARS-CoV-2 infections in Canada between January and July 2020 but only 3% by June 2021, suggesting that the measures taken at the community and institutional levels during this period effectively ameliorated the high infection rate in HCWs observed in the early epidemic [1,2]. The risk of SARS-CoV-2 infection in HCWs in Canada varied geographically, with provinces such as Quebec and Ontario showing the highest COVID-19 seroprevalence, largely due to cases in long-term care homes (LTCHs) [3–5]. Nevertheless, the onset of the first highly infectious Omicron wave in late 2021 overcame all previously effective barriers, including high vaccination rates. Over time, the rates of infection among HCWs declined relative to their community counterparts, only to increase again during the Omicron waves, mirroring what was observed in the general population. The prevalence of SARS-CoV-2 infection was nearly 60% in the general Canadian population by July 2022 [6–8].

Early in the pandemic, seroprevalence studies showed that HCWs were at higher risk of infection than community referents [9,10] due to social and health conditions, exposure to COVID-19 cases at work, and a lack of adequate protective measures [11–15]. Many of these studies were conducted in Canadian HCWs [16–19]. Large international cohort studies of HCWs have since tracked how emerging variants, vaccination and workplace policies reshape infection risk [20–27], yet comparable Canadian data on HCWs are limited [28,29] since Canadian studies have rarely included HCWs from LTCHs, predominantly focusing on the general population [6,30,31]. Only a few Canadian cross-sectional surveys have measured the burden of SARS-CoV-2 infections in LTCH HCWs [32,33], identifying risk factors for outbreaks, as well as COVID-19 incidence and associated mortality, in LTCH residents [34–37]. This scarcity of longitudinal research on HCWs creates a critical gap in understanding SARS-CoV-2 infections in a key population for pandemic preparedness.

Here, we report the establishment of a longitudinal cohort of HCWs in Southeastern Ontario, a region that maintained a low incidence of SARS-CoV-2 infections until the outbreak of the Omicron variant of concern (VOC). This presented an opportunity to explore the factors determining the relative success of epidemic control in this region, with a focus on HCWs. This work is relevant as other respiratory viruses (respiratory syncytial virus and influenza) have caused incidence peaks in the Northern Hemisphere since the winter of 2022 [38]. The cohort study recruited HCWs from one acute care hospital (ACH) and four LTCHs. Recruitment began on November 24, 2020, and ended on July 12, 2021. The cohort was followed until September 2022, allowing us to cover seven waves of the COVID-19 pandemic (S1 Table). The objectives of this report are 1) to describe the cohort in terms of distribution of main exposures, outcomes and serologic assays, 2) to describe the unadjusted incidence of COVID-19 risk in the participants, and 3) to provide a summary of the currently unpublished analysis and its relevance in addressing research gaps concerning risk of SARS-CoV-2 infection in HCWs.

### 1.1. Epidemiological and healthcare system context

The first 2 years of the COVID-19 epidemic in Ontario were marked by substantial geographical variation in incidence rates [39]. By August 30, 2022, seven COVID-19 epidemic waves had been recorded (S1 Table). During the first four waves, there was a distinct evolution of the epidemic, with a higher incidence rate (5–10 times higher) in large urban centres versus a lower one in smaller urban and suburban áreas [39,40]. This difference narrowed over time and virtually disappeared between the fifth and seventh waves, predominantly caused by the Omicron variant. The cumulative incidence of COVID-19 in Ontario by June 2022 was 8,921.2 per 100,000 inhabitants. In urban centers, such as Toronto and Ottawa, the cumulative incidence was 10,816.6 and 7,109.7 per 100,000 population, respectively. The cumulative incidence in major Ontario cities was only surpassed by that in the Northwestern region (11,592 per 100,000), which is mainly suburban/rural and overrepresents Indigenous communities. These cumulative rates contrast with that in the region where this cohort was established (6,854.7 per 100,000 population).

Provincial mandates guided the healthcare response, which presumably influenced the behaviour of HCWs in the province, a sample of whom participated in this study. S1 Fig summarizes the relevant provincial mandates and institutional regulations during the period covered by the cohort and relevant ACH policies. Briefly, in January 2020, the first case of COVID-19 was confirmed in Ontario [41]. A few weeks later, the province declared a state of emergency, with the closure of most non-essential services [42]. The vast majority of COVID-19 cases in HCWs were laboratory-confirmed by Polymerase Chain Reaction (PCR) following provincial testing guidance [43,44]. In the ACH, screening for symptomatic individuals and high-risk contacts started as early as March 2020 and remained active during the study period (ACH infection control team, personal communication). In the LTCHs, screening for symptomatic staff occurred daily province-wide, and COVID-19 testing of asymptomatic staff was performed twice a week with PCR and/or antigen-based tests from May 2020 and throughout the cohort follow-up period [45].

A universal masking rule was instituted in April 2020 for all LTCHs in the province. In the ACH, HCWs were required to wear surgical masks and eye shields when involved in the direct care of suspected/confirmed COVID-19 cases and N95 or higher-level respirators during aerosol-generating procedures (AGP), with universal masking starting in July 2020. N95 respirators became the norm for the direct care of COVID-19 cases in all healthcare facilities from Wave 5 (Omicron wave) onward (December 2021).

HCW vaccination started on December 15, 2020, for LTCH staff in higher-prevalence areas and was expanded later to regions of lower prevalence. In early March 2021, Ontario opted for a delayed second dose of the COVID-19 vaccine (4 months rather than the manufacturer-recommended 21- or 28-day intervals) and focused on administering the first dose to as many high-risk people as possible [46]. Contact tracing capacity became overwhelmed during the Omicron wave in most jurisdictions in Ontario by the end of 2021. Between January and March 2022, Ontario gradually relaxed social gathering restrictions. By mid-March 2022, outdoor masking had become optional in the province [47].

### 1.2. Cohort objectives

**Fig 1:**
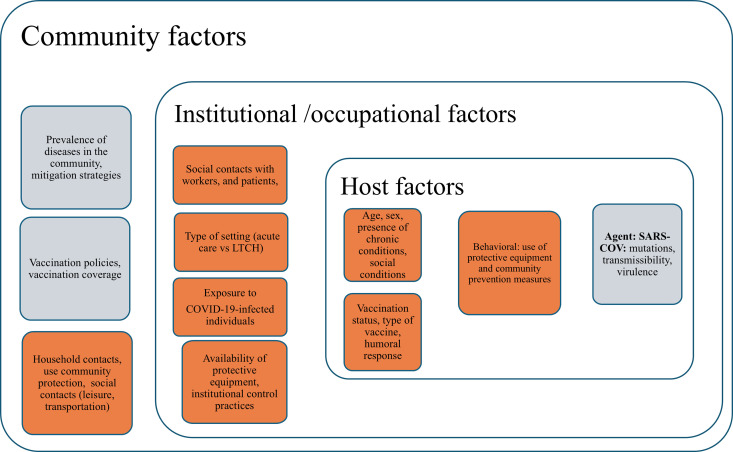
Framework to delineate risk factors for health care workers risk of COVID-19. Highlighted in orange are the factors directly assessed in the SCORE.

The SCORE (***S****erologic Evidence of* ***CO****VID-19 and Social and Occupational Contacts in Healthcare Workers in Long-Term Ca****re*** *and Acute Ca****re*** *Facilities in Southeastern Ontario)* cohort sought to describe 1) the prevalence and incidence of COVID-19 during the study period, based on self-report and serology; 2) the risk of COVID-19 from the occupational and social contact patterns of HCWs; and 3) the factors that affected the risk of acquiring COVID-19 among the cohort participants. We proposed to study the risk in HCWs by adopting the framework of the agent-host-environment triad, as depicted in Fig 1 and supported as follows.

SARS-CoV-2 is a betacoronavirus first discovered in China in December 2019. It caused a pandemic, spreading rapidly worldwide as it encountered an interconnected, fully susceptible human population. SARS-CoV-2 infects host respiratory cells via interactions between its spike (S) protein and the angiotensin-converting enzyme ACE2 on the susceptible cell surface [48]. SARS-CoV-2 has a high mutational rate, estimated at 1 × 10^−3^ substitutions per base (30 nucleotides/genome) per year or 1 × 10^−5^ to 1 × 10^−4^ substitutions per base in each transmission event [49,50], which contributed to the emergence of a multitude of variants with enhanced transmissibility and differential virulence levels and immune evasion capabilities [51]. Surveillance of SARS-CoV-2 variants throughout the pandemic was performed in most countries and has allowed detailed tracking of the evolution of this agent at the local and regional levels.

The human population was naïve to SARS-CoV-2 and experienced a wide spectrum of infection severity from asymptomatic infection to severe systemic disease and fatal respiratory failure. Factors strongly linked to higher disease severity are age, the presence of chronic conditions, and compromised immune status [9,52]. Later in the pandemic, vaccination status, vaccine type [53] and vaccine response [54,55] were found to strongly affect the clinical outcomes of SARS-CoV-2 infections. Behavioural factors, such as the use of personal protective measures in occupational and community settings (e.g., masks, social distancing and hand hygiene), acceptance of vaccines, and socioeconomic status, influenced exposure and vulnerability to SARS-CoV-2 infections [16,56,57].

The host interaction with the environment had a striking impact on SARS-CoV-2 spread. During the COVID-19 pandemic, it became clear that closer and longer social interactions and the characteristics of indoor facilities could promote SARS-CoV-2 transmission. Studies supported the associations of institution type, exposure to institutional COVID-19 cases [58], social contact [59,60] and institutional regulations. Community mitigation strategies, social contacts and the prevalence of infection in the community also influenced the risk of COVID-19 acquisition in HCWs [61]. Interactions between the host and the environment have strongly affected the infection risk of HCWs in LTCHs. Outbreaks and the risk of SARS-CoV-2 infection in HCWs have been associated with inadequate infection control practices while caring for COVID-19 patients [62,63], older age of buildings, a higher total number of beds, crowding [64] and a higher degree of interconnectedness in the facility, type of facility ownership (for-profit ownership was associated with higher risk compared with not-for-profit ownership) [36,65], and low HCW socioeconomic status [32,36,66,67]. Regional and neighbourhood social conditions are also well-established determinants of infection risk [8,68].

## 2. Methods

### 2.1. Ethics approval

This study was approved by the Queen’s Ethics Board for Research in Humans (ethics reference DEMD-2405–20). All participants provided written (electronic) consent. The consent form provided information on 1) the content and duration of the questionnaire, 2) the use of blood samples, 3) the need for monthly surveillance, and 4) the need for potential further follow-up contact. A second consent was obtained from participants to collect data during the Omicron wave, which exceeded the originally planned 9- to 12-month follow-up period. No personal identifying information was collected through the study questionnaires. HCWs received a compensation of CAD 20 after the second blood draw.

### 2.2. Cohort establishment

The SCORE cohort’s establishment started with the recruitment of HCWs in an ACH, focusing on those more likely to interact with patients with either suspected or confirmed COVID-19. The COVID-19-dedicated unit, the emergency department and the intensive care unit (ICU) were deemed high-risk settings in this regard. Subsequently, all HCWs in the ACH were invited to participate through posters and departmental emails. Additionally, 10 LTCHs were invited to participate, of which four agreed. Of these, two had experienced COVID-19 outbreaks before recruitment, and two had not. The two LTCHs that had experienced previous COVID-19 outbreaks were categorized as high-risk settings, while the two that had not were categorized as low-risk settings. In the participating LTCHs, HCWs were initially invited with the assistance of managers who forwarded email invitations and through posters that contained a QR code directing potential participants to contact the study coordinator.

Individuals were eligible to participate in the study if they were HCWs in any of the participating institutions, able to provide consent and willing to remain engaged during the 9–12-month follow-up. The surveys were completed online, except in one LTCH, where HCWs had inconsistent access to institutional e-mail and required paper-based surveys. A sample size of 200 HCWs offered sufficient power to find statistically significant differences between the prevalence of seropositivity or self-reported COVID-19 rate of 15% in settings with previous outbreaks or HCWs in high-risk ACH areas (COVID-19 unit, ICU and emergency room) and a seroprevalence or COVID-19 rate of 3% in settings with no previous outbreaks or HCWs in low-risk areas in the ACH.

Table 1 describes the five institutions included in the study. These were located in areas covered by three public health units, named Regions 1, 2 and 3 to maintain anonymity. Each region covered populations of less than 200,000 inhabitants, with two-thirds of the population living in an urban setting and Region 3 having a high proportion of indigenous communities. The ACH is the largest healthcare facility in Southeastern Ontario. Of the LTCHs included, one was located in a rural area, one was mid-sized, two were large, and two were for-profit institutions. Notably, no institutional outbreaks of COVID-19 occurred in the participating LTCHs from the start of recruitment on November 24, 2021, until the first Omicron wave. The first COVID-19 outbreak in the ACH occurred in July 2021. Further details of the LTCHs are presented in Table 1.

**Table 1:** The number of patients and staff participants in the acute care hospital and long-term care homes included in the study.

Institutional characteristics	Acute Care Hospital, ACH	LTCH 1	LTCH 2	LTCH 3	LTCH 4
**Location**	Region 1	Region 1	Region 2	Region 3	Region 1
**Low vs high risk of COVID-19 infection at the beginning of the cohort**		Low risk	High risk	High Risk	Low risk
**Size**	Large	Large	Medium	Large	LargE
**Location**	Urban	Urban	Urban	Rural	Rural
**Number beds**	440	243	60	253	174
**Ownership**		Non-profit	For-profit	Municipal	For-profit
**Local incidence before September 2020**		Low (<150 cases per 100.000)	Low (<150 cases per 100.000)	Low (<150 cases per 100.000)	Low (<150 cases per 100.000
**Cumulative incidence until June 2022 in the region**	8325,8 per 100,000	8325,8 per 100,000	5,271.0 per 100,000	5,983.3 per 100,000	8325,8 per 100,000
**COVID unit**	Yes	No	No	No	No
**Recruitment period**	24 November, 2020/ 21 June, 2021	18 February 2021/ 9 April 2021	29 December 2020/29 April 2021	15 January 2021/19 March 2021	2 June 2021/2 July 2021
**Number of participants recruited**	142	9	19	27	11
**Outbreaks since March 2020**	July 2021, Nov 2021 Jan 2022 Aug 2022	April 2022	**April 2020**; Nov 2020	**May 2020;** April 2022	Dec 2021, April 2022
**Follow-up data**	123	3	8	21	12
**Follow-up data in August/September 2022**	112	3	7	21	8

Source: Ontario LTCH database [1], analysis performed by authors.

### 2.3. Cohort follow-up

**Fig 2:**
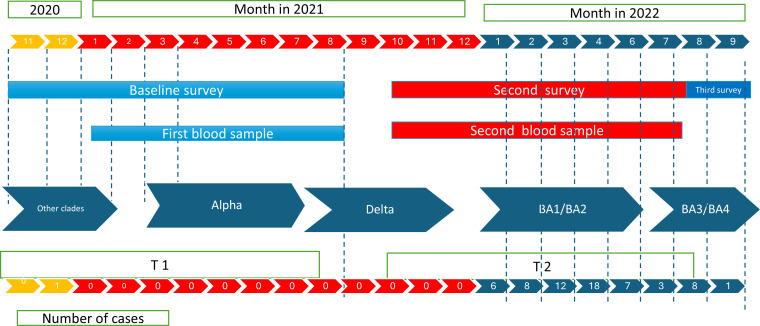
Visual description of the study by time, collection of data and number of cases in the SCORE cohort.

Data were collected using various instruments: a baseline survey, a social contact questionnaire, data abstraction of contact tracing of public health unit records, two follow-up surveys, monthly surveillance surveys, and serologic tests from two blood draws. The SCORE cohort was initially designed to include a baseline assessment (questionnaire and blood draw), a monthly questionnaire on infection, exposure and vaccination status, and a second questionnaire and blood draw 9–12 months after the initial assessment. Upon the onset of the Omicron wave, an additional survey was used to gather data during this wave. The follow-up covered Waves 1–7 of the COVID-19 epidemic in Canada (Fig 2). Enrollment in the cohort started in November 2020, and the baseline was completed in June 2021. Monthly surveys started in December 2020 and ended in July 2022. Two blood samples were collected over the study period, at the beginning of the study between January 2021 and August 2021 and 9–12 months later, between October 2021 and August 2022. The mean time between the two blood specimens was 48 weeks (range 36–80 weeks). All blood samples were collected after questionnaire completion, with a delay of 26 days (Standard Deviation (SD): 25) between the initial questionnaire and the first blood sample and 48 days (SD: 43) between the second questionnaire and the second blood sample.

### 2.4. Measurements

Data that described the factors encompassed by the agent-host-environment triad to a feasible degree, as felt pertinent to a sample of HCWs, were gathered in the SCORE cohort (Table 2).

**Table 2:** Variables and sources of data in the SCORE study.

*Category*	*Specific variables*	*Instruments*
***OUTCOME***		
*COVID-19 infection*	Ever tested for COVID-19, number of times tested, number of infections, date of infection, outcome of infection (none, search for primary care, hospitalization), self-test for COVID-19.	*Baseline, 9/12 follow-up and Post Omicron survey* *Monthly short survey*
*Seroprevalence of COVID-19*	Positivity, negative, undetermined	*Abbot test assessed at two-time points.*
*Humoral immunity*	Levels of IgG two times over the follow-up; qualitative assessment: positive, negative, undetermined	*Euroimmune test assessed at two-time points.*
***HOST FACTORS***		
*Social determinants*	age, sex, postal code, minority status in Canada, migration history, income sufficiency, size of the household, and number of rooms.	*Baseline, 9/12 follow-up and Post Omicron survey*
*Comorbidities and smoking status*	List of chronic conditions and diagnoses of any new condition over the follow-up.	*Baseline, 9/12 follow-up and Post Omicron survey*
*Source of infection*	Date of a positive test, relation of the case to an outbreak, family contact, work contacts, source of contact by place; number of contacts located, traced and informed	*Contact tracing data from public health units*
*Symptoms and severity of infection*	List of symptoms	*Contact tracing data from public health units*
*COVID-19 Vaccines*	Date of each vaccine and type of vaccine received	*Baseline, 9/12 follow-up and Post Omicron survey* *Monthly short survey*
***ENVIRONMENT***		
*Occupational risk factors*	Exposure to an outbreak at the institution, job role, number of confirmed/suspected COVID-19 patients exposed to, use of personal protective equipment (PPE), exposure to aerosol-generating procedures; floor visited during work, work location within each facility.	*Baseline, 9/12 follow-up and Post Omicron survey* *Monthly short survey*
*Household factors*	Number and age of people at home, exposure to COVID-19 in household members, number of vaccinated household members.	*Monthly short survey*
*Social contacts at work and in the community*	Gender, sex, place of contact, frequency of contact and closeness of contacts in the last 24 hours, number of contacts with patients over a day, duration of contact with patients; contact with coworkers (duration and number), locations of work in institution, time spend in each place in each institution	*Social contact survey*
*Community risk exposures*	Exposure to non-occupational outbreaks, type of setting (bar, party, religious meeting etc)	*Monthly short survey*
*Use of preventive measures in the community*	Frequency of use of a mask, hand sanitizer and social distancing (6 feet or more apart) when outside.	*Baseline, 9/12 follow-up and Post Omicron survey*

**The primary outcome was SARS-CoV-2 infection**, determined by a self-reported positive test for COVID-19. All LTCH HCWs in the province of Ontario were tested for SARS-CoV-2 infection using PCR testing when symptomatic or screened weekly or biweekly with either PCR or antigen-based tests when asymptomatic (between May 2020 and June 2023) [69]. HCWs at the ACH were required to undergo PCR-based COVID-19 testing when symptomatic or suspected to have had close contact with a COVID-19 case (Infection Control Office of the ACH, personal communication). Participants recorded the exact date of the positive test, which was used to define the outcome. Importantly, HCWs could access their COVID-19 test results and vaccination history via the Ministry of Health’s COVID-19 registry.

#### Assessment of humoral response.

Three antibody tests were used to detect previous SARS-CoV-2 infections [70–72]. The first test was the Abbott SARS-CoV-2 IgG assay (Abbott Diagnostics, Abbott Park, Illinois, United States), which detects IgG antibodies against the nucleocapsid (N) protein of SARS-CoV-2. For this test, a relative light unit index (S/C) with a result of > 1.4 was considered positive for past COVID-19. This assay has modest sensitivity (approximately 70%) but high specificity (≥ 90%) [73]. The second test was the semiquantitative Euroimmun Anti-SARS-CoV-2 ELISA IgG (Medizinische Labor Diagnostika AG, Lübeck, Germany), which detects IgG antibodies against the SARS-CoV-2 S1 domain of the S protein, including the immunologically relevant receptor-binding domain. This assay offers three possible interpretations: negative if the ratio is < 0.8, borderline if the ratio is ≥ 0.8 to 1.1 and positive if the ratio is ≥ 1.1. The latter assay was used during the first phase of recruitment but was abandoned when vaccination started because of cross-reactivity with vaccination-induced antibodies and because it did not allow the quantification of high antibody levels expected to be induced by vaccination. A third ELISA test was used to quantify IgG antibodies against the SARS-CoV-2 S protein’s receptor–binding domain (EUROIMMUN, product number: EI 2606-9601-10) [74]. This quantitative method offers a linear range between 3.2 and 384 binding antibody units (BAU)/mL. Samples with results over 384 BAU/mL were diluted by a factor of 20–30 to obtain numeric results. These dilutions typically rendered diluted concentrations that fell within the optimal range of quantitation of this assay. The actual antibody concentration was calculated by multiplying the detected concentration by the dilution factor. A cut-off of 35.2 BAU/mL was used to determine seroconversion, as recommended by the manufacturer [70]. The antibody testing was performed in the acute care hospital’s clinical laboratory, where established training and standard procedures are in place to ensure the accuracy of test results. The same laboratory technologists consistently followed standard procedures when dilution was needed.

**Factors related to the risk among HCWs in each setting** were selected based on the available literature and our framework (Fig 1). These included 1) host-related factors, such as sociodemographic variables, health-related variables and variables related to COVID-19 vaccines; 2) occupational variables, including setting, job role, use of protective measures, exposure to COVID-19 cases at work, exposure to AGP, and number of contacts at work; 3) household factors, including number and age of people at home and vaccination status of the household members; and 4) community-related factors, including contact during leisure, school and transportation activities, exposure to cases in community settings, and use of protective measures in the community. Further details of all variables included and how they were collected are available in the questionnaires, presented as supplementary materials.

### 2.5. Analysis

First, a descriptive profile of the cohort was generated, reporting means ± SD, ranges and frequency/percentage distributions for all key exposures, outcomes and serological results. Group comparisons used bivariate statistics tailored to variable type, including χ² or Fisher’s exact tests for categorical measures. These comparisons were applied 1) across clinical settings (ACH vs. LTCHs) and 2) between participants who remained in follow-up versus those lost to follow-up, allowing assessment of potential selection bias. To examine incident SARS-CoV-2 infections over time, a discrete-time hazard model with logit links was employed, producing an interval-specific odds ratio. Calendar time was partitioned into five epochs that aligned with the major Canadian epidemic waves (First three waves: Delta, early Omicron and Omicron BA.4/5). Person periods were censored at the earliest infection, withdrawal or study end. All analyses were conducted in Stata 15 (StataCorp, College Station, TX, USA).

## 3. Results

### 3.1. Cohort Description

#### Cohort participation and retention.

**Fig 3:**
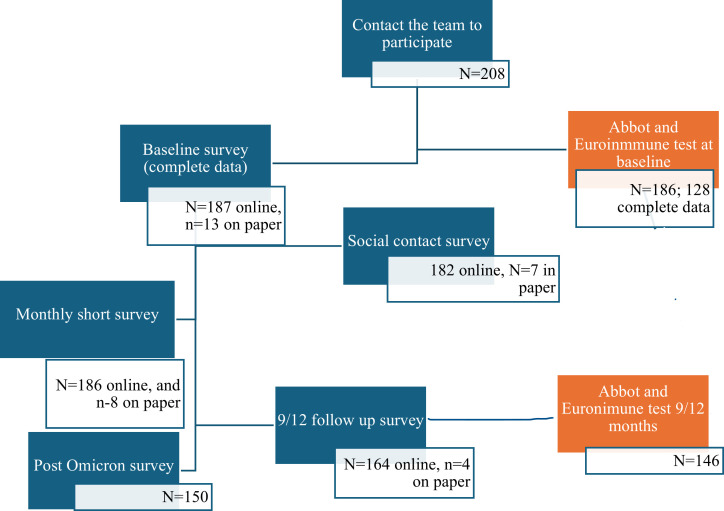
Recruitment and specimen collection of the SCORE cohort.

The cohort recruited 208 HCWs who agreed to participate, of whom 200 provided complete baseline data, 168 (81%) provided two data points between May 2020 and July 2022, and 150 (72%) provided an additional (third) data point between August and September 2022 (Fig 3). The cohort retention rate was 72%. The main reasons for loss to follow-up included relocating to another city and changing work sites. Participants who were lost to follow-up were more likely to be nurse assistants or personal support workers (PSWs), report less sufficient income, and be smokers. More participant loss occurred in the LTCHs than in the ACH (S2 Table).

### 3.2. Participant characteristics

Sixty-eight percent of the sample originated from the ACH. Most respondents were women (85%), with an average age of 37 years. Nearly 60% of the sample comprised nurses, nurse assistants and PSWs (Table 3). Thirty-six percent had at least one chronic condition, and 10% smoked tobacco. Most (90%) were born in Canada, and 14% self-identified as a visible minority (based on religion, race or gender). The majority of HCWs (66.8%) reported not having any difficulty meeting household needs, with the rate being lower among HCWs from LTCHs. Differences in demographic characteristics were observed between HCWs in the ACH versus the LTCHs, with the former being younger, more economically self-sufficient, having a lower smoking rate and fewer chronic conditions, and living in urban areas (Table 3).

**Table 3:** Baseline sociodemographic characteristics of the participants in the SCORE, total and by settings.

Variable/characteristics	Total, N = 208	Acute Care Center, ACH, n = 142	LTCH/low risk n = 20	LTCH/ high risk n = 46	P value (differences across settings)
**Occupation**					P < 0.001
Physician	22, 10.9	20,14.6	1, 5.3	1, 2.3	
Nurse/NP	91, 45.1	71, 51.8	7, 36.8	13, 28.3	
Nursing assistant (nurse aid,/PSW, patient attendant)	29, 14.4	3, 2.2	7, 36.8	19, 41.3	
Therapists (physiotherapist, respiratory therapist)	16, 7.9	14, 17.2	--	2, 4.4	
Laboratory/radiology technicians, phlebotomists,	17, 8.4	17, 12.4	--	--	
OHCW(social worker, dietary worker, pharmacist, nutritionist)	13, 6.4	4, 2.9	2, 10.5	7, 15.2	
Support/social services (ward clerk, housekeeper, administrator, research assistant)	14, 6.93	8, 5.84	2, 10.53	4, 8.70	
**Age, average, SD**	37.4; 11.1	36.2; 10.2	39.7; 9.9	39.8; 13.5	0.001
Less than 30	25, 12.2	16,11.4	1, 5.26	8, 17.4	
30-39	87,42.4	65, 46.4	8, 42.1	14,30.4	
40-49	42, 20.5	33, 23.5	2, 10.5	7,15.2	
50-59	30, 14.6	15,10.7	8, 42.1	7, 15.2	
60 and more	21,10.24	15,10.7	0	10,21.7	
**Sex**					0.72
Female	123, 85.1	117, 83.7	17, 89.4	40, 86.9	
Difficulty meeting household needs- *					0.001
Very difficult	4, 1.95	–	1, 5.3	3, 65	
Fairly difficult	7, 3.41	3, 2.14	2, 10.5	2, 4.4	
A little difficult	46, 22.4	25, 17.8	8, 42.1	13, 28.3	
Not at all difficult	137,66.8	107, 76.4	6, 31.6	24, 52.2	
Prefer not to answer	11, 5.37	5, 3.57	2, 10.5	4, 8.7	
**Born in Canada, yes**	185, 90.2	126, 90.0	16, 84.2	43, 93.4	0.51
**Visible minority, yes**	28,13.4	20, 14.1	3, 15.0	5, 10.9	0.83
**Have a chronic condition.**					0.36
No	136, 65.4	99, 69.7	11, 55.0	26, 56.5	
1	42, 20.2	24, 16.9	5, 25.0	13, 28.2	
2 or more	30, 14.4	19, 13.3	4, 20.0	7, 15.2	
**Current smokers**	21, 10.4	7, 5.15	4, 21.0	10, 21.7	0.008
**Living arrangements**					
Living alone	43, 20.9	36, 25.7	1, 5.3	6, 13.04	0.05
Living with children (<18yo)	67, 32.2	49, 34.5	7, 35.0	11, 23.9	0.39
Living with older adults (60 and more)	15, 7.2	8, 5.6	3, 15.0	4, 8.7	0.28
Number of persons at home, mean; Standard deviation	1.77; 1.45	2.32;1.2	2.11; 1.1	2.23;1.3	0.28
Living in a rural area	42,20.8	20, 14.1	6, 31.6	16, 39.0	0.001

* Considering your household income, how difficult is it for you and your family to meet your monthly housing-related costs? Housing costs include rent/mortgage, property taxes and utilities only. Would you say that it is...

### 3.3. Prevalence and incidence over time

COVID-19 cases in the SCORE cohort had a bimodal distribution (Tables 4 and 5), with cases reported by HCWs at the beginning of the pandemic and during the Omicron waves. A total of 16 of 206 (7.6%) HCWs reported having had COVID-19-positive tests between March and July 2020. During the monthly follow-up, only three participants reported a positive COVID-19 test, one in November 2020, one in December 2020 and one in January 2021. New cases started to be reported again in January 2022, with a peak in cases between March 2022 and June 2022. A total of five reinfections were identified in the SCORE cohort. Two participants had both of their infections before the Omicron waves, two participants had one infection pre-Omicron and another during the Omicron waves, and one participant reported two infections during the Omicron waves (further details in S3 Table).

**Table 4:** Estimated hazards using survival discrete analysis expressed as percentages and odds ratios in the whole sample and by setting.

Interval in months	Hazard (%) (logit OR) Whole sample	Hazard %(logit OR) ACH sample	Hazard %(logit OR) LTCH Low risk	Hazard %(logit OR) LTCH high risk
February 2020 – June 2020 (Wave one)	6.25 (ref)	1.41 (ref)	0	23.9 (ref)
July 2020 - November 2020 (Wave one and two)	1.03 (OR=0.15)*	1.43 (OR=1.01)	0	0
December 2020 – April 2021 (wave two)	1.04 (OR=0.16)*	1.45(OR=1.02)	0	0
May 2021 – September 2021 (Delta)	0	0	0	0
October 2021-February 2022 (Omicron)	8.23 (OR=1.34)	7.56 (OR=5.7)*	0	14.3 (OR=0.53)
March 2022- July 2022 (BA.1/BA.2)	37.31 (OR=8.9)*	42.0 (OR=50.7)*	18.2	26.1 (OR=1.12)
August – September 2022 (BA.4/BA.5)	1.23 (OR=0.18)	1.75 (OR=1.25)	0	0

* Denote p value<0.05; OR= odds ratio.

**Table 5:** Calculated hazards using survival discrete analysis expressed as percentages and odds ratios in the acute care center.

Interval in months	Hazard %(logit OR) ACH sample/ high risk	Hazard %(logit OR) ACH sample/ low risk
February 2020 – June 2020 (Wave one)	0	1.98 (ref)
July 2020 - November 2020 (Wave one and two)	2.44 (ref)	1.01 (OR=0.50)
December 2020 – April 2021 (wave two)	0	2.04 (OR=1.03)
May 2021 – September 2021 (Delta)	0	0-
October 2021-February 2022 (Omicron)	0	10.3(OR=5.7)*
March 2022- July 2022	37.9 (OR=24.4)*	43.6 (OR=38.3)*
(BA.1/BA.2)	0	2.56 (OR=1.30)

* Denote p value<0.05; OR= odds ratio.

HCWs from the LTCHs at high risk had a higher rate of infection at the beginning of the pandemic than those in the ACH (23.9% vs. 1.41%). HCWs from the ACH had a higher risk of infection during the Omicron waves than during the preceding waves (Odds Ratio (OR) = 7.64; CI95%: 4.24–13.7), while in the LTCHs at high risk, HCWs experienced a similar risk of infection before and during the Omicron waves (OR = 1.76; CI95%: 0.63–4.9). For those in LTCHs at low risk, the odds ratio was not calculated as there were no cases before Omicron (Table 4), and new cases occurred only later between March and July 2022. In HCWs who had worked in high-risk settings in the ACH, the hazard of COVID-19 was low over the follow-up period until the beginning of March 2022, when the hazard was 37% (Table 5).

Regional genotype surveillance depicted the transition of SARS-CoV-2 variants from the beginning of the epidemic onward (S2 Fig). The distribution of the predominant sublineages in the three regions mirrored those reported in the province of Ontario, where sublineages were identified as predominantly BA.1/BA.2 between January 2 and June 18, 2022, and BA.4/BA.5 between June 19 and November 26, 2022. Assuming infections in this cohort by the reported date, they resembled the pattern seen in Ontario. Overall, 17% of the cases were due to non-Omicron lineages, 68.4% occurred during BA.1/BA2 predominance, and 14.4% occurred when BA4/BA5 was predominant.

### 3.4. Occupational exposures over time

At baseline, 8% of the cohort had worked in a COVID-19 unit, 12% in the emergency room and 20% in the ICU. During the Omicron wave, 11% worked in a COVID-19 unit, 24% in the emergency room and 34% in the ICU. At baseline, 50% of the participants had provided care to a COVID-19 case, which rose to 67.9% during the Omicron wave. Nearly 18% of HCWs reported caring for more than 20 COVID-19-confirmed patients at baseline, which rose to 38% during the Omicron wave. In the ACH, HCWs in the high-risk area maintained a similar percentage of exposure to cases over the study period, but those working in low-risk areas had an increase in exposure to COVID-19 cases at work. A similar pattern was observed in HCWs from the LTCHs at low risk. The LTCHs at high risk had the lowest proportion of exposure to cases at work during the Omicron waves (Table 6).

**Table 6:** Description of the cohort in terms of risk exposure to COVID-19. Occupational and community exposures.

	During the first year of the epidemic (at 9, and 17 months), N, %	During 2021 N, %	Since November 2021 N, (%)
**Exposure to COVID-19 cases (confirmed) in institution**			
Total	105, 50.5	95, 56.5	106, 67.9
**ACH**	69, 48.6	81, 65.8	83, 72.2
High-risk (ICU, COVID-19 unit, or emergency room)	61, 85.9	51, 92.7	51, 92.8
Low risk	8, 11.2	30, 53.3	32, 54.3
**LTCH low risk**	0, 0	7, 63.6	8, 72.7
**LTCH high risk**	36, 78.2	7, 20.5	15, 50.0
**Exposure to COVID-19 cases at home**	8, 3.85	16, 9.5	60, 40.8
**Practice the use of masks in the community (most of the time/always)**	180, 94.2	158, 94.0	44, 30,0
**Practice social distancing in the community (most of the time/always)**	83, 43.2	123, 73,2	69. 47.2
**Practice washing hands in the community, (most of the time/always)**	142,74.3	147, 87.4	114, 78.5

A higher proportion of HCWs (94%) used N95 respirators during the Omicron wave than earlier in the epidemic (47%), while the use of surgical masks was reduced when providing care to COVID-19 patients (S3 Fig), being replaced by the use of N95 or higher-level respirators. At the beginning of the epidemic, some HCWs from the ACH were already using N95 respirators, although this was not the case for HCWs in the LTCHs. This trend changed over time so that 80% of HCWs used N95 respirators when caring for COVID-19 patients/residents (S4 Fig).

### 3.5. Household and community exposures

Exposure to household COVID-19 cases was also higher during the Omicron waves (Table 6). This was more evident in ACH HCWs, with five reported cases of COVID-19 at home pre-Omicron and 50 reported cases at home during the Omicron waves (data not shown). In addition, there was a shift in behaviours throughout the observational period: HCWs reported a decreased frequency of use of protective measures during community interactions, specifically reduced mask use and social distancing. This behavioural change occurred between the late 2021 and early 2022 periods, consistent with the provincial relaxation of community measures in Ontario in March 2022.

### 3.6. Vaccination history

By the end of 2020, Health Canada had approved six COVID-19 vaccines, but three were predominantly used: Moderna SpikeVax (mRNA, mRNA-1273), Pfizer-BioNTech Comirnaty (mRNA, BNT162b2) and AstraZeneca Vaxzevria (viral vector-based, AZD1222). The majority of the cohort (95%) received Pfizer as the first dose, 95% received it as the second dose, and 89% received it as the third dose. Moderna was the second most frequently received vaccine, with 4% for the second dose and 10.8% for the third dose. AstraZeneca was received by two HCWs as the first dose and by one HCW as the second dose.

Vaccination in the cohort started in December 2020. At baseline, 46% of the HCWs had been vaccinated (21.6% of the ACH HCWs, 57% of the HCWs in LTCHs at low risk, and 46% of the HCWs in the LTCHs at high risk; Table 7), reflecting vaccine prioritization among HCWs and residents of LTCHs [75]. In Ontario, administration of the first booster COVID-19 vaccination started in November 2021 for HCWs responding to increasing Omicron cases. At the beginning of the first Omicron wave, 15% of the cohort had received one dose, 80% had received two doses, and 3% had received three doses. During the Omicron wave, 65% received a third dose. By the end of August 2022, 99% of HCWs in the ACH were vaccinated; the corresponding percentages were 88.9% in the LTCHs at low risk and 97% in the LTCHs at high risk (Table 7). The time interval between the first and second doses ranged from 4 to 40 weeks, with a mean of 15 weeks. The mean time between the second dose and the booster (third dose) was 26 weeks, with a range of 5–78 weeks (more details in S5 Fig). In Canada, the approved interval between the second dose and the booster was ≥ 6 months for immunocompetent individuals.

**Table 7:** Characteristic of the cohort sample of HCWs by analytical samples for T1 and T2.

Variable/characteristics at baseline (first survey)	Sample at T1 January–August 2021	Sample at T2 October 2021–August 2022
**ACH**	N = 83	N = 103
**Antibody levels,** BAU/mL, mean, range	66.7; 3.2-766.8	1888.06; 10.5-10787.1
**Reported infection before blood collection, N, %**	3, 3.61	10, 9.71*
**Time since last infection and antibody assessment, mean; SD**	24 weeks; 18 weeks	8.7 weeks,: 6.3
**Qualitative anti-N IgG antibody assay, N, %**	2, 2.41	5, 4.90
**Number of vaccines before blood collection, N, %**		
None	55, 66.3	1, 1.0
One	28, 33.7	0, 0
Two		39, 37.8
Three		60, 58.2
Four		3, 2.91
**LTCHs low risk**	N = 12	N = 9
**Antibody levels,** BAU/mL, mean, range	1956.45; 399-5320.9	1265.5; 361-5603.4
**Reported infection before blood collection, N, %**	0, 0%	1, 11.1%*
**Time since infection and antibody assessment**	--	2 weeks
**Qualitative anti-N IgG antibody assay, N, %**	0, 0%	1, 11.11
**Number of vaccines before blood collection, N%**		
None	2, 16.7	
One	9, 75.0	
Two	1, 8.3	
Three		9, 100
Four		
**LTCHs high risk**	N = 33	N = 30
**Antibody levels,** BAU/mL	1634.5; 3.2-6654.6	2250.5; 373- 10508.1
**Reported infection before blood collection, N,%**	9, 27.3	7 23.3*
**Time since infection and antibody assessment, mean, SD**	55.7 weeks, 4.4 weeks	10.4 weeks, 5 weeks
**Qualitative anti-N IgG antibody assay, N, %**	3, 9.1	3, 10.0
**Number of vaccines before blood collection, N, %**		
None	15, 45.4	0,0
One	12, 36.4	1,3.3
Two	6, 18.2	3, 10.0
Three		25, 83.3
Four		1, 3.3

*exclude infections acquired before T1.

### 3.7. Humoral response

A total of 182 and 145 HCWs provided data on anti-N antibody titres at T1 (first blood sample) and T2 (second blood sample), respectively. Five participants had positive anti-N antibody titers at T1, three of whom reported no previous positive COVID-19 PCR test. At T2, 10 HCWs were positive for anti-N antibody titers, two of whom did not report a previous positive COVID-19 PCR test. This suggests that five cases might have had asymptomatic or minimally symptomatic COVID-19 that did not prompt testing.

In 177 HCWs at T1 and 146 at T2, we could assess qualitative IgG titres: 59.3% were positive at T1 and 99.3% were positive at T2 (> 1.1). Due to logistical limitations, 56 samples at T1 were discarded before the SARS-CoV-2 anti-S protein quantitative IgG antibody assay could be performed. All these samples had rendered negative results with the semiquantitative anti-S protein IgG antibody assay. In the participants who had IgG quantitation, the mean levels of anti-S IgG were 648 BAU/mL at T1 (n = 128) and 1,913 BAU/mL at T2 (n = 142). The IgG antibody levels ranged between 3.2 BAU/mL and 6,654 BAU/mL at T1 and between 10.5 BAU/mL and 10,787 BAU/mL at T2. There was no correlation between IgG levels at T1 and T2 (Spearman rank test r = 0.03; p = 0.72); thus, levels at T1 did not predict levels at T2.

**Fig 4:**
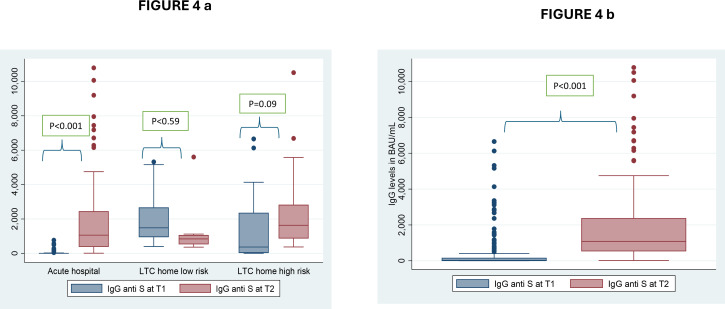
Distribution of anti-S IgG antibody concentration in HCW at two-time points. (4a) Distribution of anti-S IgG antibody concentration in HCW at two-time points and by facility type using non-parametric paired analysis, there was an increase in antibody levels in the acute hospital HCW and in those from the LTC homes at high risk. (4b) Distribution of anti-S IgG antibody concentration in HCW at two-time points in the whole sample. Differences calculated using Wilcoxon signed-rank test.

In HCWs from the ACH, an increase in IgG levels was observed between T1 and T2, which was not observed in HCWs from the LTCHs (Table 7 and Fig 4a). Differences in IgG levels were observed between institutions at T1 (p < 0.001), with HCWs at the LTCHs having higher IgG levels than those in the ACH. Those differences were not observed at T2 (p = 0.07), which likely reflects that HCWs in the ACH were less likely to be vaccinated at T1 while being more exposed to infection (3.6% ➔ 9.7%) and vaccines at T2 (two/three-dose coverage climbed from 0% to 96%; Fig 4b and Table 7).

## 4. Discussion

The SCORE cohort offers opportunities to deeply explore the agent, host, and environmental factors influencing SARS-CoV-2 transmission in Ontario-based HCWs. In the context of the COVID-19 epidemic in Ontario, the SCORE cohort reflects the evolution of the epidemic in Southeastern Ontario, a small city, semi-urban and rural setting. S6 and S7 Figs describe the cases since the start of the epidemic in the three public health districts where the participating institutions are located. We observed a low incidence of COVID-19 cases until the onset of the first Omicron wave, which highlighted the drastic impact of this VOC on transmission and the importance of infectious agent characteristics. Our analysis indicated a ninefold increased risk of infection compared to earlier periods of the pandemic.

Remarkably, the ACH was outbreak-free for 16 months, and the low-risk LTCHs remained so for at least 24 months from the beginning of the pandemic in Ontario. This was the case even with the emergence of more infectious Alpha and Delta variants in early 2021, with essentially no reported incident cases in the cohort during that time. Only after the Omicron wave arrived, did outbreaks and cases occur in these facilities. These preliminary results suggest that institutional and community strategies were effective at maintaining the HCWs at low risk until the Omicron waves.

Risk factors for COVID-19 infection with a focus on occupational and community factors have been examined [76]. Given the distribution of hazards over time, we could assess the associations between occupational and community exposures and infection risk in two different periods, at the beginning of the pandemic and during the Omicron waves. Very few studies in HCWs have been able to perform these longitudinal comparisons [53,77].

Importantly, our preliminary analysis of hazard risk over the study period suggested that working in the ACH high-risk areas did not confer a higher risk of acquiring COVID-19, neither at the beginning of the epidemic nor during the Omicron waves. HCWs in low-risk areas of the ACH started to be exposed to COVID-19 cases during the Omicron waves, when institutional outbreaks became frequent in this facility. Exposure to cases at work has been related to an increased risk of infections when protective equipment and infection control measures were insufficient [4]. Overall, in this cohort, the risk of SARS-CoV-2 infection in HCWs declined once these interventions were put in place, which was more obvious in the LTCH HCWs. Our multivariate analysis has confirmed that exposure to institutional COVID-19 cases resulted in a differential effect before (increased risk of infection) and during (no association with risk of infection) the Omicron waves [76].

In Canada, HCWs from LTCHs were disproportionately affected by COVID-19 during the first year of the epidemic [2,5,9,78]. In our sample, HCWs from high-risk LTCHs had a high risk of infection in the early epidemic, followed by no cases during Waves 3 and 4 and few cases during the Omicron waves. HCWs from low-risk LTCHs started to report infections in March 2022. Although our sample of LTCH HCWs is small, several observations could explain these findings: protective equipment, such as N95 respirators, was not used by HCWs in the LTCHs but was used by some HCWs at the ACH, as described in other Canadian studies [2,79]. LTCH HCWs were nurses and auxiliary workers who had closer contact with residents. Finally, the LTCH HCWs had lower socioeconomic status than the ACH HCWs overall. Other studies have highlighted important risk factors among HCWs in LTCHs. These include higher social (low payment and stability) and health (stress and fatigue) vulnerability of the HCWs and their patients (older and with more infectiousness), type and frequency of close contact with residents, lack of availability and appropriate use of PPE, and facility conditions [34]. Unfortunately, we did not have a sample large enough to evaluate factors that determined higher or lower risk across the LTCH facilities and LTCH HCWs [80]. The virtual absence of incident COVID-19 in our cohort of LTCH HCWs may have in part been due to COVID-19 vaccination, which likely contributed to the low risk of infection until March 2022.

The community environment played an important role in the risk among HCWs, which could be further explored in SCORE. The low prevalence in the region where the cohort was established was likely related to the strict mitigation measures in the community, such as school closures, gathering restrictions, masking, and social distancing mandates (S1 Fig). Those measures were relaxed in March 2022, a period in which we observed a surge of infections in the cohort. The risk of infection in this cohort coincided to a certain degree with community prevalence and household cases: few household cases in the early pandemic and a large increase in household cases during Omicron were noted. Earlier in the pandemic and during Omicron, household exposure to COVID-19 cases had correlated with incident infection in HCWs in other studies [16], an aspect that was confirmed in a multivariate analysis of the SCORE cohort data [76]. The increase in exposure to COVID-19 cases at home during the Omicron waves is consistent with the increase in cases in the community, including among children [38]. Since 35% of the HCWs resided with children, this is an important aspect to assess in future analysis. Children were reported to be effective transmitters of SARS-CoV-2 during the Omicron waves for several reasons: most children were naïve to previous immune events (either infection or vaccination) from school closures, which limited their exposure to COVID-19 cases earlier in the pandemic, and COVID-19 vaccination coincided with the onset of the fifth COVID-19 wave (first Omicron wave).

Furthermore, we observed less frequent use of community preventive measures, such as wearing a mask and distancing, in the community. Many factors could have contributed to this: the perception of low risk due to previous infection or vaccination, the psychological fatigue due to the long duration of mitigation mandates, and likely government relaxation of measures [81,82]. There is also evidence of the low effectiveness of masking in the community against the Omicron variants [83]. In a recent analysis of the SCORE data, we tested associations between the use of protective measures in the community and the risk of infection before and during the Omicron period. Our findings supported that in both periods, the use of protective measures was related to a lower infection risk [76].

Antibody assays can offer a glimpse into SARS-CoV-2 vaccine-mediated versus infection-mediated humoral responses and the chance to explore surrogates of protection [54,55]. Anti-SARS-CoV-2 IgG antibody levels in HCWs have been rarely quantified in Canada [84,85]. Therefore, our results could be compared or aggregated with data from other studies as needed [30,86,87]. We observed an increase in the levels of anti-S protein IgG antibodies at T2 compared to T1, which may relate to more events of infection and/or vaccination in the cohort. At T2, nearly 90% of the cohort had received two vaccine doses, and nearly 12% of HCWs had had a previous Omicron infection before T2. IgG levels achieved in the participants are difficult to compare with other data, as the assays varied across studies [88]. Omicron infections tended to induce weaker immune responses compared to other variants, and mild infections tended to cause a less robust immune response. Using Poisson regression to assess associations for highly skewed distributions [89], we will establish the relationships between the number of vaccine doses and previous (yes/no) infection regarding anti-S IgG antibody levels, testing the hypothesis that hybrid immunity is associated with higher antibody levels of this type, which has been reported elsewhere in Canada [30]. We will also determine whether the timing of vaccination and infection, as well as demographic and health factors, are related to higher antibody levels. The SCORE study also offers the chance to compare antibody levels achieved before and during the Omicron waves in a specific population of HCWs.

Furthermore, we collected information on social contacts in two different ways. First, we used the Polymod survey [90–92] to gather data about contact type, duration and frequency. Second, we obtained contact tracing data. Unfortunately, contact tracing data could no longer be recorded after December 2021 in Ontario public health units, as the Omicron waves overwhelmed contact tracing capacity. The social contact information will be used to determine the number of social contacts, contact risk level and duration, and contact types associated with a higher risk of COVID-19. Additionally, differences in social networks can be explored via egocentric analysis of social contacts in participants with and without COVID-19. Networks could also be compared across different settings (ACH vs. LTCHs) and may allow comparisons with other Canadian studies [93].

### 4.1. Strengths and weaknesses

Although other cohorts of HCWs in Canada during the COVID-19 pandemic have been established [16,28,94], the SCORE study is unique in several ways. A sizable number of HCWs from various disciplines, roles and practice types, including nurses, physicians, nursing assistants/PSWs, respiratory therapists and laboratory technicians, were included, whereas other studies mainly recruited physicians. One other study with a sizable LTCH HCW population published only cross-sectional data, but no study on this population group has published longitudinal data [5]. No study in Canada has previously sampled LTCH HCWs, assessed their occupational and household risk factors, and longitudinally followed them through the COVID-19 epidemic.

The SCORE study incorporates detailed surveys of occupational and non-occupational factors. For instance, we inquired about work site, intra-facility mobility, and contact frequency and type (with patients and other HCWs), which allowed analyses of mobility, contact variability and risk of COVID-19 [95]. Further, we measured social contact, which can affect the risk of COVID-19 acquisition, by adapting questions from the Polymod survey to assess closeness, duration and number of community and household contacts [96]. These data are comparable to those obtained in another Canadian cohort [93]. Lastly, the SCORE study collected serum samples to measure SARS-CoV-2 anti-S protein IgG antibodies during the pre-Omicron and Omicron waves.

The SCORE study needed some adjustments throughout the epidemic to respond to new knowledge and the urgent deployment of COVID-19 vaccination. We adjusted the surveys to collect data on the number of doses, vaccine type and timing, and incident SARS-CoV-2 infections. This resulted in advantages and limitations. For instance, the type of vaccine was not assessed in the first survey, as it was not clear when and what vaccines were to be deployed. Once vaccines became available for deployment [97], the vaccine type was inquired about in the second survey. As a result, at this later time point, responses were more prone to memory bias, and missing information was more frequent for the second vaccine dose. We mitigated this limitation by reconstructing probable vaccination dates using the monthly short survey, which offered an acceptable yet modestly imprecise alternative. Importantly, the number of vaccine doses and the last dose proved to be more reliable than their timing. Despite these limitations, the obtained descriptive data revealed vaccine timing consistent with the recommended provincial vaccination recommendations.

We are confident that the study captured the vast majority, if not all, COVID-19 cases, since all HCWs with symptoms of COVID-19 were required to undergo confirmatory PCR testing. We may have missed some asymptomatic infections, which have been reported at a rate of 7.3% or lower in a different study [98]. For this, we assessed the presence of SARS-CoV-2 anti-N antibodies and identified three unreported infections at baseline and two during the Omicron waves. These cases will be considered as incident COVID-19 cases in future analyses. Despite the limited sensitivity of serologic assays to identify COVID-19 infections, anti-N antibody detection proved useful for this purpose. This is affected by time since infection [99]; thus, there is a chance of false negative results. Pooled analysis has rendered a sensitivity of 8l% and a specificity of 97.0% with ELISA-based assays [100]. We will exert caution when interpreting antibody levels by considering the health characteristics of the population and the severity of infections [101]. We have created a causal diagram to control for confounders in the analysis of IgG levels that could inform our and other studies (S8 Fig).

This is a convenience sample of HCWs with low participation, likely less than 5%. The participation rate from LTCHs as the invited population varied over time due to the temporary suspension of on-site activities of healthcare trainees and the inability to confirm the number of people who were effectively invited via e-mail. Participation rates of HCWs in cohort studies have been reported to be low, especially in Ontario [28], at 4% for physicians and 18% for nurses. Thus, the characteristics and proportions of social and occupational parameters cannot be generalized to other HCWs of the participating institutions. The high burden of HCWs in caring for residents with COVID-19 infection, the strenuous circumstances of social isolation, enhanced COVID-19 surveillance, and the effects of school closures on families presumably contributed to the low participation in the study.

Carazo et al. [5] studied HCWs in Quebec, with a sample of 2,085 HCWs from LTCHs, and revealed a similar profile of participants to that found in the SCORE cohort: low physician representation, a high proportion of nurses and health care support workers, and the overrepresentation of women. While they did not report data on the social conditions of HCWs, other studies have shown an overrepresentation of immigrants and low-income HCWs in LTCHs, as in SCORE [32,102]. Nevertheless, the SCORE data resemble the trends in COVID-19 in the regions where the facilities are located. Data from the first three digits of the participants’ postal codes could also be used to explore community characteristics and infection risk, as others have done [68].

Unfortunately, 20% of the sample was lost before the assessment of the second survey, possibly biasing the results regarding the Omicron waves. Loss to follow-up was related to income, smoking, and job role, factors that are also related to COVID-19. Therefore, those who remained in the study were at a lower risk of infection. Moreover, the characteristics of those lost to follow-up resulted in the underrepresentation of the cohort in terms of LTCH workers. Studies among HCWs in LTCHs have shown high rates of occupation change, less stability, and sickness, consistent with reported causes of missing information for the second and third surveys. Attrition rates in another Canadian study were lower at 11%, but attrition rates higher than or closer to those in our study have been reported as problematic during the COVID-19 pandemic [103]. We will use causal diagrams to describe the source of selection bias in SCORE and establish the best method to quantify and correct it [104].

## 5. Conclusions

This paper presents a comprehensive overview of the SCORE cohort—its design, instruments, participant profile and principal findings—so that other researchers can readily incorporate its longitudinal HCW data into future collaborative studies [93]. Two broad insights emerge for regions that began the pandemic with low SARS-CoV-2 prevalence. First, the arrival of VOCs, especially Omicron, sharply increased infection risk even in a highly vaccinated workforce, underscoring how viral evolution can outpace existing immunity. Second, the epidemic’s trajectory in HCWs was shaped by opposing trends in infection control strategies: ACH and LTCHs progressively strengthened protective measures, while community-level restrictions were eased, highlighting the interplay between institutional policies and broader public health interventions. The authors welcome and encourage research collaborations using the SCORE data. Data are available on reasonable request, and researchers are welcome to contact the research group for further information.

## Supporting information

S1 Table The waves of COVID-19 in Canada and SCORE study assessments. (DOCX)

S2 Table Descriptive data and sociodemographic characteristics of participants with complete vs incomplete follow-up. (DOCX)

S3 Table Reinfections in the cohort of HCW-SCORE 2020–2022. (S3 table.DOCX)

S1 Fig Provincial Mandates and Institutional Regulations During The Period Covered By The SCORE Cohort. (DOCX)

S2 Fig This is fig title Percent SARS-CoV-2 lineages circulating in Eastern Ontario 12/27/2020 to 08/30/2022. (DOCX)

S3 Fig This is fig title: Personal protective equipment used when caring for COVID-19 patients by follow-up period. This is figure 3 legend: Figure 3a corresponds to the proportion of HCW using PPE at beginning of the pandemic, figure 3b depicts PPE during 2021 and figure 3c PPE during Omicron wave.
(DOCX)

S4 Fig Personal protective equipment used when caring for COVID-19 patients by period and facility type. This is figure 4 legend: Figure 4a corresponds to the beginning of the pandemic, figure 4b, during 2021 and figure 4c during the Omicron wave.
(DOCX)

S5 Fig Distribution of timing of vaccine doses since the beginning of epidemic (Feb. 1^st^ 2020) in weeks.
(DOCX)

S6 Fig Laboratory confirmed COVID-19 weekly case counts and rates reported in Ontario, January 12, 2020, to June, 2022 (DOCX)

S7 Fig Laboratory confirmed COVID-19 weekly case counts and rates by reported date in the regions where the HCW were recruited, January 12, 2020, to December 31, 2022. (DOCX)

S8 Fig Causal model for the analysis of antibody levels. This is figure 8 legend: According to the casual model 1. In testing the relationship between sociodemographic variables on IgG at T1, there is no need oto adjust for vaccines or infection. 2. In testing the relationship between sociodemographic variables on IgG at T2, there is no need oto adjust for any other variable. 3. To estimate the effecto of vaccines and infection on IgG at T1, we need to adjust for sociodemographic and health variables, 4. To estimate the effecto of vaccines and infection on IgG at T2, we need to adjust for sociodemographic and health variables and IgG at T1, 5. In testing the relationship between IgG at T2 and infection after T2, we need to adjust by vaccine and infection before T2.
(DOCX)
