# Supplementary material for: SCORE: Serologic evidence of COVID-19 and social and occupational contacts in healthcare workers in long-term care and acute care facilities in Southeastern Ontario (SCORE)
Source: PLoS One. 2025 Aug 13;20(8):e0303813. doi: 10.1371/journal.pone.0303813 (PMC12349196; doi:10.1371/journal.pone.0303813)
Supplement: S1 Table — (DOCX) [file pone.0303813.s001.docx]

**The waves of COVID-19 in Canada and SCORE study assessments**

| **Wave** | **Dates** | **SCORE study assessment that covered this period** |
| --- | --- | --- |
| Wave one (175 days) | February 2020–July 2020 | Baseline survey |
| Wave two (230 days) | July 2020–Mid–March 2021 | Baseline and monthly short survey |
| Wave three (140 days)  VOC - Alpha | March 2021–July 2021 | Monthly short survey |
| Wave fourth (104 days)  VOC – Delta | July 2021-November 2021 | Monthly short survey and 9/12 month survey |
| Wave fifth (117 days)-VOC - Omicron | November-2021- Feb 2022 | Monthly short survey and 9/12 survey |
| Subsequent waves | March 2022- | Monthly short survey, 9/12 month survey, Post-Omicron survey |
