# Supplementary material for: SCORE: Serologic evidence of COVID-19 and social and occupational contacts in healthcare workers in long-term care and acute care facilities in Southeastern Ontario (SCORE)
Source: PLoS One. 2025 Aug 13;20(8):e0303813. doi: 10.1371/journal.pone.0303813 (PMC12349196; doi:10.1371/journal.pone.0303813)
Supplement: S2 Table — (DOCX) [file pone.0303813.s002.docx]

**Descriptive data and sociodemographic characteristics of participants with complete vs incomplete follow-up**

| **Variable/characteristics** | **Baseline sample, n, %** | **Sample with three assessments, %** | **Sample missing survey data, n, %** |
| --- | --- | --- | --- |
| **Setting** |  |  |  |
| Acute care center | 142, 68.3 | 109, 74.1* | 33, 54.1 |
| LTC low risk | 20, 9.6 | 10, 6.8 | 10, 16.39 |
| LTC high risk | 46, 22.1 | 28, 19.0 | 18, 29.51 |
| **Profession** |  |  |  |
| Physician | 22, 10.89 | 20, 13.9* | 2, 3.45 |
| Nurse/NP | 91, 45.05 | 66, 45.8 | 25, 43.1 |
| Nursing assistant/PSW | 29, 14.36 | 14, 9.7 | 15, 25.86 |
| Therapists | 16, 7.92 | 12, 8.3 | 4, 6.90 |
| Lab technicians | 17, 8.42 | 13, 9.0 | 4, 6.90 |
| OHCW | 13, 6.44 | 11, 7.6 | 2, 3.45 |
| Support/social services | 14, 6.93 | 8, 5.6 | 6, 10.34 |
| **Age, average, SD** | 37.3(11.08) | 38.2(11.3) | 35.32(10.08) |
| **Sex** |  |  |  |
| Women | 123, 85.1 | 123, 84.2 | 51, 83.3 |
| **Income *** |  |  |  |
| Not at all difficult | 137, 66.8 | 106, 72.6* | 31, 52.54 |
| **Born in Canada** | 185, 90.2 | 131, 89.7 | 54, 91.5 |
| **No chronic condition** | 136, 64.9 | 100, 68.0 | 36, 54.1 |
| **Current smokers** | 21, 10.4 | 12, 6.8* | 9, 16.7 |

* differences between those with and without loss of follow-up significant at level p<=0.05
