# Supplementary material for: SCORE: Serologic evidence of COVID-19 and social and occupational contacts in healthcare workers in long-term care and acute care facilities in Southeastern Ontario (SCORE)
Source: PLoS One. 2025 Aug 13;20(8):e0303813. doi: 10.1371/journal.pone.0303813 (PMC12349196; doi:10.1371/journal.pone.0303813)
Supplement: S3 Table — (S3 table.DOCX) [file pone.0303813.s003.docx]

**Reinfections in the cohort of HCW-SCORE 2020-2022.**

| Participant | **Date of first infection** | **Date of second infection** | **Antibody levels at T1** | **Antibody levels at T2** |
| --- | --- | --- | --- | --- |
| 1 | March 2020 | April 2022 | No assessed | 6940 BAU/ml |
| 2 | 26 November 2020 | 1 July 2021 | 92 BAU/ml | 632 BAU/ml |
| 3 | 15 April 2020 | 14 July 2021 | 3373 BAU/ml | No assessed |
| 4 | 3 March 2022 | 12 august2022 | No assessed | No assessed |
| 5 | At Lab T1- May 2021 | At lab T2- April 2022 | 1318 | 224 BAU/ml |
