# Supplementary material for: SCORE: Serologic evidence of COVID-19 and social and occupational contacts in healthcare workers in long-term care and acute care facilities in Southeastern Ontario (SCORE)
Source: PLoS One. 2025 Aug 13;20(8):e0303813. doi: 10.1371/journal.pone.0303813 (PMC12349196; doi:10.1371/journal.pone.0303813)
Supplement: S1 Fig — (DOCX) [file pone.0303813.s004.docx]

**Provincial Mandates and Institutional Regulations During The Period Covered By The SCORE Cohort**

Feb 3 2020

Screening symptomatic visitors

staff residents

Ref 1

April 8

Universal masking LTC

April 14 2020 LTC staff restricted to work at one single facility

Ref 7

April 22,2020

PCR testing surveillance all staff LTC

Ref 3a 3b

Dec 14 2020

COVID-19 vaccination starts in with LTC HCW and residents

Ref 4

Jan-Mar 2022

Provincial Relaxation of restrictions

Mar 17 2022 outdoor masks become optional

Dec 23 2021

Ontario recommends a third dose vaccine for priority groups including HCW

Ref 6

Mar 13 2020

Visitors to LTC residents restricted to essential visitors

Ref 1

2021

Mar 13 2020

Droplet contact precautions for the routine care of COVID-19 cases

Personal communication

SCORE cohort recruitment and follow-up period

With few exceptions most HCW received the 2 first vaccine doses 4 months apart Ref 5

July 14 2020

Universal masking in acute care hospital

Aug

2022

2020

| Long-Term Care Facilities |
| --- |
| Feb  Acute-care hospital |

Nov 2021

Second Covid-19 outbreak in acute care hospital

July 5 2021

First Covid-19 outbreak in acute care hospital

Jan- Feb 2020

PCR testing capacity building in Ontario

Ref 2

May 2020

COVID-19 unit created to provide care to all cases COVID-19 patients not needing ventilatory support

Personal communication

Ref 1

https://www.ontario.ca/page/covid-19-action-plan-long-term-care-homes#section-2

Ref 2

<https://www.publichealthontario.ca/en/About/News/2020/Story-Covid-19-Testing-Ontario>

Ref 3

<https://www.ontario.ca/page/covid-19-action-plan-long-term-care-homes>

<https://toronto.ctvnews.ca/ontario-to-begin-proactive-testing-for-all-long-term-care-residents-one-month-into-covid-19-outbreak-1.4906755?cache=%2Fparliament-where-decorum-and-diplomacy-went-to-die-1.2429094>

Ref 4

<https://news.ontario.ca/en/release/59607/ontario-begins-rollout-of-covid-19-vaccine>

Ref 5

<https://www.publichealthontario.ca/-/media/Documents/nCoV/Archives/Vaccine-Uptake/2021/04/covid-19-vaccine-uptake-report-2021-04-22.pdf?rev=1921bd22031d4ed08ef6b509d71e4686&la=fr>

Ref 6

<https://www.publichealthontario.ca/-/media/Documents/nCoV/Vaccines/2021/12/covid19-vaccine-recommendations-omicron-variant.pdf?rev=67d321a75ec947909c979bc895b91a11&sc_lang=en>

Ref 7

<https://news.ontario.ca/en/release/56681/ontario-ramping-up-protection-for-long-term-care-residents>

(ltc staff to work at one single facility)

<https://jammi.utpjournals.press/doi/full/10.3138/jammi-2020-0027> (this article found that in an acute care hospital only 0.2% of HCW were + when tested for covid-19. N approx. 5K.
