# Supplementary material for: SCORE: Serologic evidence of COVID-19 and social and occupational contacts in healthcare workers in long-term care and acute care facilities in Southeastern Ontario (SCORE)
Source: PLoS One. 2025 Aug 13;20(8):e0303813. doi: 10.1371/journal.pone.0303813 (PMC12349196; doi:10.1371/journal.pone.0303813)
Supplement: S2 Fig — (DOCX) [file pone.0303813.s005.docx]

**Percent SARS-CoV-2 lineages circulating in Eastern Ontario 12/27/2020 to 08/30/2022**


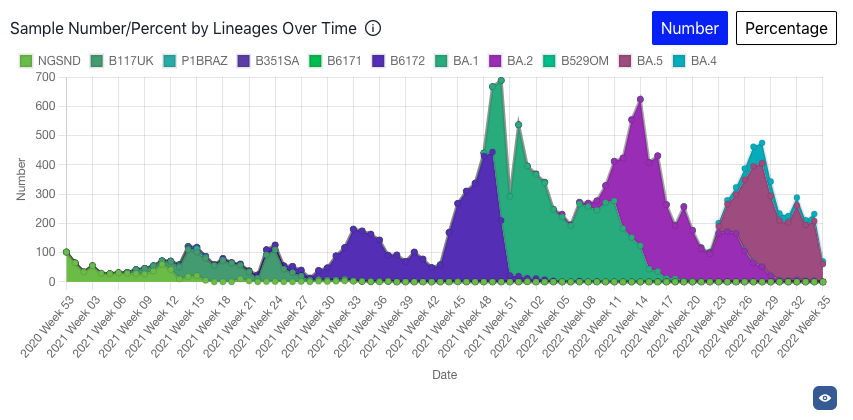


**Source: KHSC Clinical Microbiology Dashboard SARS-CoV-2 sequencing**
