# Supplementary material for: SCORE: Serologic evidence of COVID-19 and social and occupational contacts in healthcare workers in long-term care and acute care facilities in Southeastern Ontario (SCORE)
Source: PLoS One. 2025 Aug 13;20(8):e0303813. doi: 10.1371/journal.pone.0303813 (PMC12349196; doi:10.1371/journal.pone.0303813)
Supplement: S3 Fig — This is figure 3 legend: Figure 3a corresponds to the proportion of HCW using PPE at beginning of the pandemic, figure 3b depicts PPE during 2021 and figure 3c PPE during Omicron wave. (DOCX) [file pone.0303813.s006.docx]

**Personal protective equipment used when caring for COVID-19 patients by follow-up period**

***
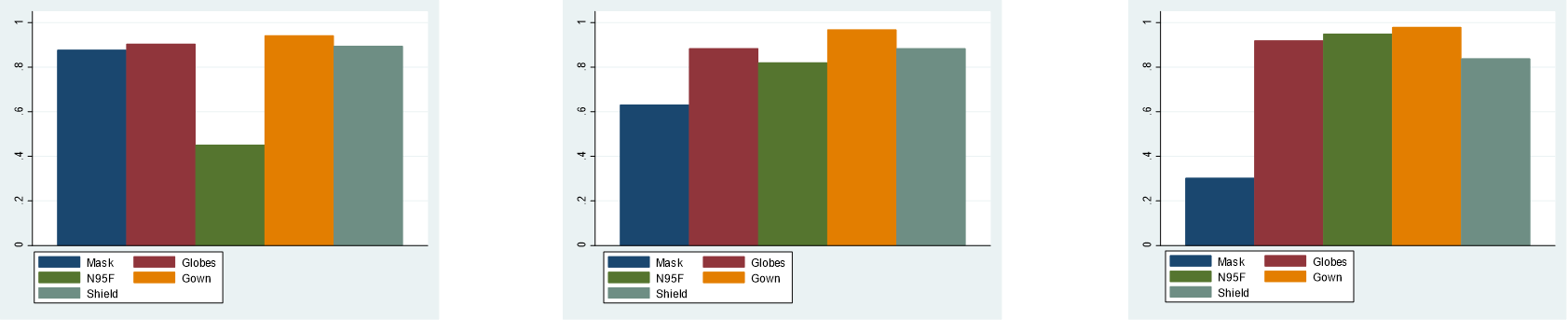
***Figure 3a corresponds to the proportion of HCW using PPE at beginning of the pandemic, figure 3b depicts PPE during 2021 and figure 3c PPE during Omicron wave.

3c

3b

3a
