# Supplementary material for: SCORE: Serologic evidence of COVID-19 and social and occupational contacts in healthcare workers in long-term care and acute care facilities in Southeastern Ontario (SCORE)
Source: PLoS One. 2025 Aug 13;20(8):e0303813. doi: 10.1371/journal.pone.0303813 (PMC12349196; doi:10.1371/journal.pone.0303813)
Supplement: S4 Fig — This is figure 4 legend: Figure 4a corresponds to the beginning of the pandemic, figure 4b, during 2021 and figure 4c during the Omicron wave. (DOCX) [file pone.0303813.s007.docx]

**. Personal protective equipment used when caring for COVID-19 patients by period and facility type.** Figure 4a corresponds to the beginning of the pandemic, figure 4b, during 2021 and figure c during the Omicron wave.

***
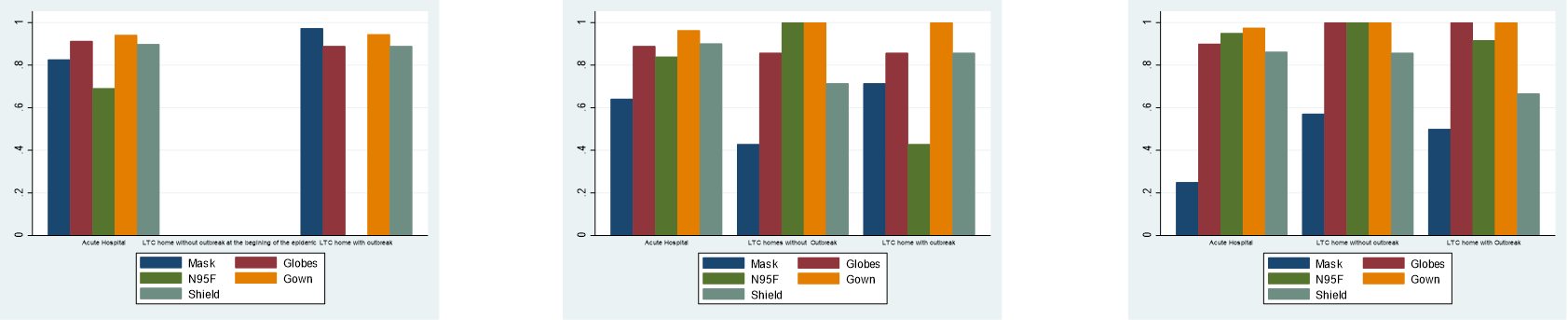
***
