# Supplementary material for: SCORE: Serologic evidence of COVID-19 and social and occupational contacts in healthcare workers in long-term care and acute care facilities in Southeastern Ontario (SCORE)
Source: PLoS One. 2025 Aug 13;20(8):e0303813. doi: 10.1371/journal.pone.0303813 (PMC12349196; doi:10.1371/journal.pone.0303813)
Supplement: S5 Fig — 1st 2020) in weeks. (DOCX) [file pone.0303813.s008.docx]

**Distribution of timing of vaccine doses since the beginning of epidemic (Feb. 1^st^ 2020) in weeks.**

2022

2021
