# Supplementary material for: SCORE: Serologic evidence of COVID-19 and social and occupational contacts in healthcare workers in long-term care and acute care facilities in Southeastern Ontario (SCORE)
Source: PLoS One. 2025 Aug 13;20(8):e0303813. doi: 10.1371/journal.pone.0303813 (PMC12349196; doi:10.1371/journal.pone.0303813)
Supplement: S6 Fig — (DOCX) [file pone.0303813.s009.docx]

**Laboratory confirmed COVID-19 weekly case counts and rates by reported date in Ontario, January 2020, to June, 2022**
