# Supplementary figures and images for: SCORE: Serologic evidence of COVID-19 and social and occupational contacts in healthcare workers in long-term care and acute care facilities in Southeastern Ontario (SCORE)
Source: PLoS One. 2025 Aug 13;20(8):e0303813. doi: 10.1371/journal.pone.0303813 (PMC12349196; doi:10.1371/journal.pone.0303813)

**Causal model for the analysis of antibody levels**


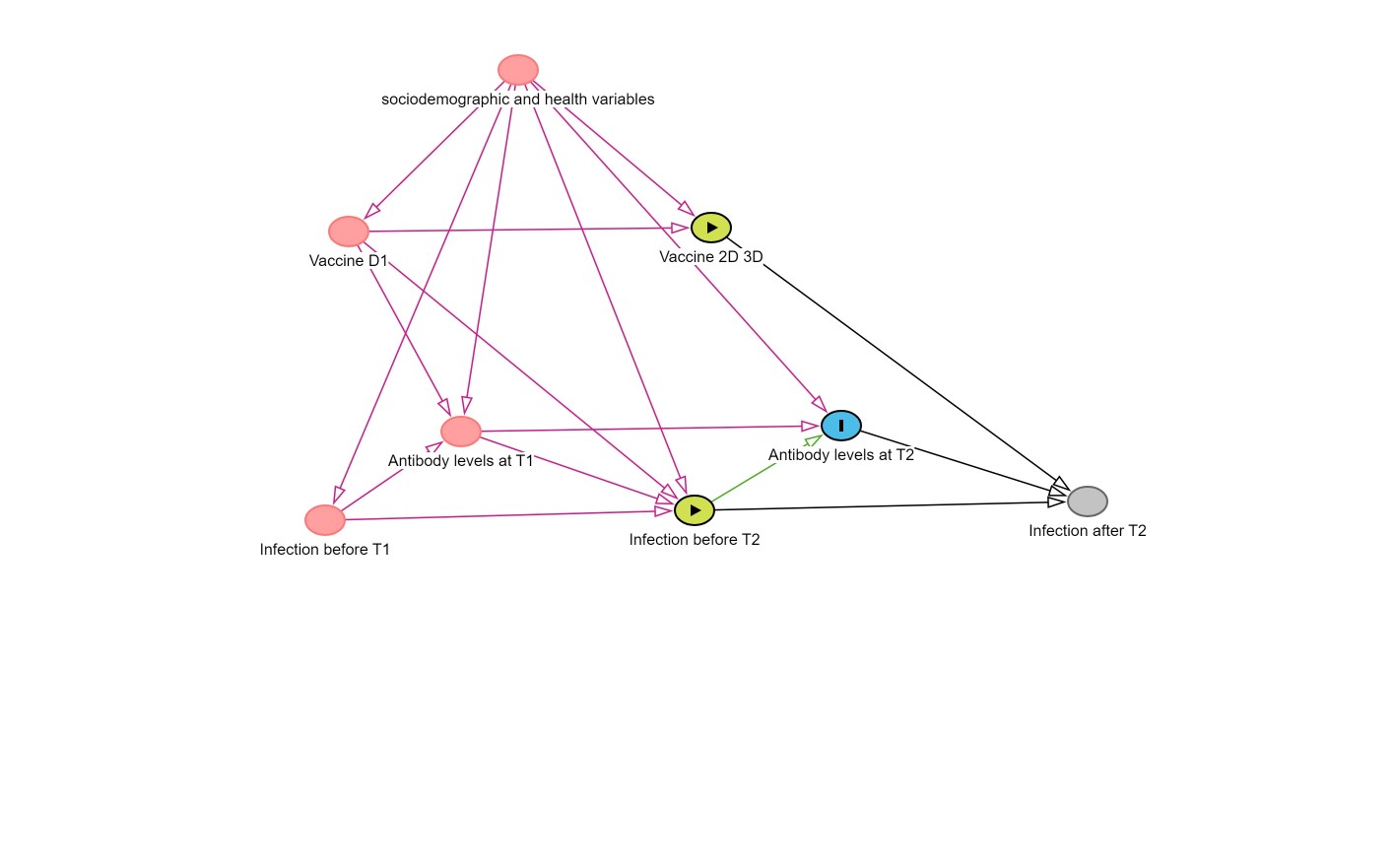


https:/dagitty.net/mScHrbdvD

Supplement: S8 Fig — This is figure 8 legend: According to the casual model 1. In testing the relationship between sociodemographic variables on IgG at T1, there is no need oto adjust for vaccines or infection. 2. In testing the relationship between sociodemographic variables on IgG at T2, there is no need oto adjust for any other variable. 3. To estimate the effecto of vaccines and infection on IgG at T1, we need to adjust for sociodemographic and health variables, 4. To estimate the effecto of vaccines and infection on IgG at T2, we need to adjust for sociodemographic and health variables and IgG at T1, 5. In testing the relationship between IgG at T2 and infection after T2, we need to adjust by vaccine and infection before T2. (DOCX) [file pone.0303813.s011.docx]
